# Salivary biomarkers in non-invasive oral cancer diagnostics: a comprehensive review

**DOI:** 10.1590/1678-7757-2024-0151

**Published:** 2024-09-09

**Authors:** Ravina VATS, Pooja YADAV, Afsareen BANO, Sapna WADHWA, Rashmi BHARDWAJ

**Affiliations:** 1 Maharshi Dayanand University Centre for Medical Biotechnology Rohtak Haryana India Maharshi Dayanand University, Centre for Medical Biotechnology, Rohtak, Haryana, India

**Keywords:** Oral Squamous Cell Carcinoma, Saliva, Non-invasive diagnostics, Tobacco consumers, Biomarkers, Oral Cancer

## Abstract

**Objective:**

This review aims to provide a comprehensive analysis of the effectiveness of saliva as a non-invasive diagnostic marker for oral cancer. Despite progress in oral cancer diagnosis and prognosis, the 5-year survival rate remains low due to the resistance to treatment and delayed diagnosis, which can be attributed to various factors including tobacco and alcohol consumption, genetic damage, and human papillomavirus (HPV). The potential use of saliva as an easily accessible non-invasive screening and diagnostic method arises from its direct contact with the lesion site.

**Methodology:**

Data for this study were gathered via a comprehensive literature evaluation using search engines such as the PubMed, Web of Science, Google Scholar, and SciFinder.

**Results:**

Identifying salivary biomarkers shows potential to transform oral cancer diagnostics by offering a reliable alternative to the traditional invasive methods. Saliva is an abundant reservoir for both cell-bound and cell-free organic and inorganic constituents. Thus, saliva is an appropriate field for research in proteomics, genomics, metagenomics, and metabolomics.

**Conclusion:**

This review provides a comprehensive elucidation of salivary biomarkers and their function in non-invasive oral cancer diagnosis, demonstrating their potential to enhance patient outcomes and reduce the impact of this devastating disease.

## Graphical abstract

**Figure:**
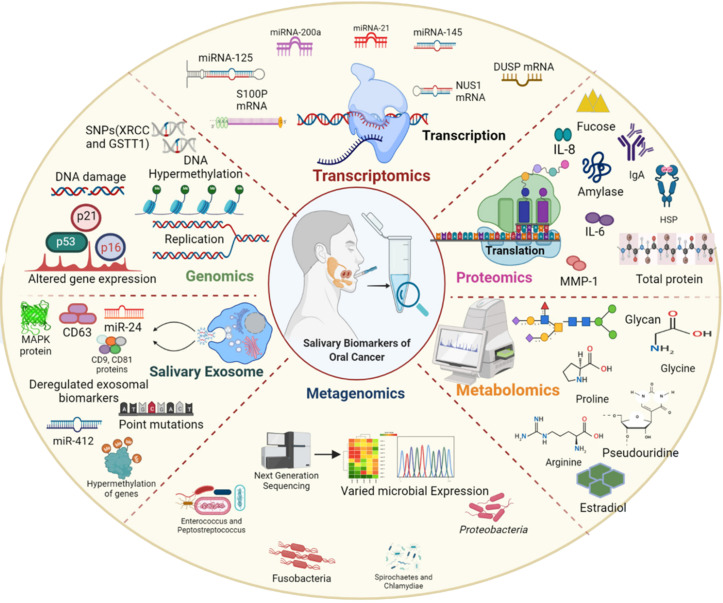

## Introduction

Oral cancer is characterized by the presence of malignant cells in the oral cavity, which comprises the sublingual space, tongue, lip, soft and hard palates, and buccal mucosa. Oral Squamous Cell Carcinoma (OSCC) originates from squamous cells in the oral cavity and accounts for nearly 90% of all oral malignancies.^1^ According to a publication issued by the World Health Organization, 377,713 newly diagnosed cases and 177,757 deaths were reported worldwide by 2020, indicating the prevalence of this type of cancer.^2^ Oral cancer significantly burdens public health and economic resources, primarily due to its high risk of mortality and morbidity, low five-year survival rate, delayed diagnosis, and the continuous emergence of numerous new cases each year.^3^ Oral cancer is primarily attributed to genetic modifications that occur at both minor and significant levels, such as point mutations involving base insertions, deletions, and substitutions as well as DNA rearrangements encompassing translocation, inversion, deletion, and duplication. Various risk factors, such as human papillomavirus (HPV) infection, tobacco use, smoking, betel quid chewing, and alcohol consumption significantly contributes to the onset and progression of oral cancer.^4^ These carcinogens can induce mutations and changes in DNA methylation, acetylation, ubiquitination, and histone modifications at the promoter sites of tumor suppressor genes and oncogenes, thereby initiating various malignancies.

Despite numerous advancements in therapeutics and preventive measures, the diagnostic approach to oral cancer still needs improvement, as traditional techniques are non-invasive and evaluate cancer at advanced stages.^5^ The existing diagnostic methods for oral cancer include visual examination, toluidine dye staining, exfoliative cytology, biopsy, and fluorescence imaging. Tissue biopsy is the gold standard for diagnosing oral cancer, but its invasive nature makes patients apprehensive, which can lead to delays in diagnosis. The uncomfortable nature of the extraction process and delayed diagnosis, which contribute to a high mortality rate, are the primary drawbacks of these screening methods.^6^ Therefore, it is crucial to identify early predictive biomarkers to enhance the survival of patients living with oral cancer. Biomarkers are molecular entities produced from endogenous genes and exhibit a substantial increase in expression in neoplastic cells. In contrast, these entities may manifest as novel gene products, typically inactive in healthy cells, but capable of regulating the expression of oncogenes and tumor suppressor genes. Biological markers can aid in predicting disease incidence rates, facilitating diagnosis and prognosis, and enabling the monitoring of treatment responses in individuals affected by the disease. Based on the preceding alterations, biomarkers are categorized into several groups: genetic biomarkers, which involve alterations at the gene level; protein biomarkers, which include the discovery of cancer-specific antigens; metabolic biomarkers, which involve the identification of altered metabolites; and metagenomic biomarkers, which detect changes in the microbial genome of the organ and may lead to uncontrolled proliferation, increased vascularization, and altered metabolism.^7^ Biomarkers for oral cancer can detect changes in DNA, RNA, and protein synthesis as well as in microRNAs, extracellular vesicles, circulating tumor cells, metabolites, and oral microbiota. Oral cancer biomarkers can be used to measure risk, diagnosis, prognostic stage, therapy response, and disease recurrence.

Oral cancer biomarkers can be studied in various body fluids. However, saliva, a susceptible and non-invasive diagnostic fluid that directly interacts with oral cancer lesions, is being considered for screening and diagnosis.^3^ The capacity of saliva to convey health information reflects that of the human body. Significant developments have been made in technologies designed to measure and analyze small amounts of analytes, including proteins and mRNA. Thus, saliva can be regarded as the circulatory system in the oral cavity. The heterogeneous saliva has been found to be slightly acidic, containing water, proteins, lipids, electrolytes, inorganic elements, DNA and RNA.^8^ Nucleic acids in saliva may originate locally within the oral cavity or be derived from serum. The examination of salivary biomarkers has been shown as capable of detecting changes in organs located distally from the oral cavity, extending beyond the realm of oral illnesses. This information includes many molecular details that could link the disease prognosis and diagnosis across various body systems.^9^

Examining saliva provides a wide range of opportunities for scholarly investigation and practical application in both elementary and clinical contexts. The discovery of salivary biomarkers has demonstrated the potential to enhance survival rate by offering a dependable indicator of both the initial and advanced stages of the disease at different intervals. This facilitates the evaluation of drug resistance and the probability of tumor recurrence.^10^ Moreover, owing to its ease of collection, transportation, storage, and sample volume, saliva shows several advantages over serum and tissue.^11^ Due to this accessibility and wide range, salivary biomarkers play a more favorable role than invasive procedures in the screening and detection of oral cancer. This review provides a detailed overview of salivary biomarkers and their roles in non-invasive oral cancer diagnostics.

Data for this review was extracted from the PubMed, Science Direct, NIH, and Google Scholar databases using search terms, including “saliva,” “non-invasive,” “biomarkers,” and “oral cancer.” Studies included in the review focused on detecting oral cancer biomarkers present in saliva. Additionally, the research emphasized the interpretation of common oral cancer biomarkers from non-invasive and minimally invasive sample sources. Studies that solely employed invasive methods of oral cancer diagnostics were excluded from the review.

### Salivaomics: Salivary biomarkers in oral cancer

Salivaomics, a sophisticated approach within the field of “omics” encompasses five diagnostic components: genetics, transcriptomics, proteomics, metabolomics, and microbiomics. The submandibular, parotid, and sublingual glands produce whole saliva, a unique blend of water, organic compounds, and inorganic molecules. Additionally, the buccal, labial, lingual, and palatal glands contribute to salivary production. The tubarial gland, a newly discovered salivary gland, may reduce the side effects of radiotherapy as revealed using prostate-specific membrane antigen (PSMA) positron emission tomography/computed tomography (PET/CT) imaging.^12^ Water, organic compounds, and inorganic components are absorbed by salivary glands from the bloodstream and integrated into their secretions. Therefore, saliva is referred to as a “window of health status,” as substances identified in plasma can also be detected in whole saliva.^13^ Saliva enables mastication, oral hygiene, phonation, digestion, homeostasis, pathogen management, enzymatic digestion, and growth suppression.^14^ Saliva has garnered significant attention as a diagnostic fluid that is non-invasive and easily collectible. Saliva can be classified into two distinct categories, stimulated and unstimulated. Several methods are available for stimulating salivary production, including masticatory activity and chewing gum. Unstimulated saliva can be obtained without mechanical, masticatory, or external gustatory stimuli.^15^ Without stimulation, the salivary flow rate and quality are affected by medications and therapy, disease, and physiological and psychological factors.

Salivary biomarkers can either originate locally or be derived from serum, both of which contribute to their presence in the saliva.^16^ Serum-derived compounds are generated via both transcellular and paracellular pathways. The transcellular pathway utilizes active and passive transport, whereas the paracellular route involves salivary gland-blood capillary ultrafiltration. The salivary glands are surrounded by highly permeable blood capillaries. This characteristic facilitates the transfer of molecules between the bloodstream and saliva.^17^ Thus, saliva contains indicators that can convey health status, potentially serving as a non-invasive alternative to blood and other invasive methods. Moreover, saliva can serve as a viable substitute for serum in the diagnosis of specific conditions. The importance of saliva in diagnostics has significantly increased due to recent technological advancements. Salivary biomarkers have been employed for screening and detecting numerous malignancies, including oral cancer, periodontal disease, dental caries, viral infections such as HIV, infectious diseases, autoimmune disorders, and drug abuse monitoring.^11^

Being non-invasive and in close proximity to lesions related to oral cancer, saliva can serve as a diagnostic tool for the identification of oral cancer. Studies have found cell-free DNA, altered DNA, RNA, proteins, metabolites, and microbial communities in the saliva.^18^ There are significant disparities between the physiological characteristics of the normal and cancerous tissues. Cell-free DNA, RNA, and other waste from apoptotic and necrotic cells in healthy settings are phagocytosed. Under pathological conditions, this mechanism is compromised, causing cellular accumulation in physiological fluids and in tissue microenvironments. Oral cancer tumors contain cell-free DNA and RNA in saliva. Cancerous cells and necrotic bodies present a larger cell-free DNA size range (100–400 base pairs) than apoptotic cells (180–200 base pairs). This phenomenon can be attributed to the enhanced metabolic activity of the malignant cells, which subsequently leads to necrosis. Together with serum-derived findings, there is growing evidence for the release of proteins and nucleic acids from apoptotic, necrotic, and malignant cells.^19^ Additionally, exosomes contain these compounds. Vesicles released by live cells and exosomes are membrane-bound and range in diameter from 30 nm to 150 nm. Cell-free molecules are crucial for the interplay between cells because they include proteins, messenger RNAs (mRNAs), and microRNAs (miRNAs).^20^Given the presence of many biomarkers in saliva, it has the potential to be used as a diagnostic medium for the detection of oral cancer. A comprehensive discussion of the numerous components of salivaomics and the specific applications of these components as oral cancer diagnostic biomarkers is presented in subsequent paragraphs.

Salivaomics provides a complex and non-invasive diagnostic method by analyzing genetics, transcriptomics, proteomics, metabolomics, and microbiomics. This method demonstrates substantial promise for the early identification of oral cancer and other disorders, as saliva contains a multitude of indicators. The subsequent paragraphs delve into a comprehensive examination of these elements and their utilization in diagnosing various conditions.

### Salivary genomics

**Figure 1:**
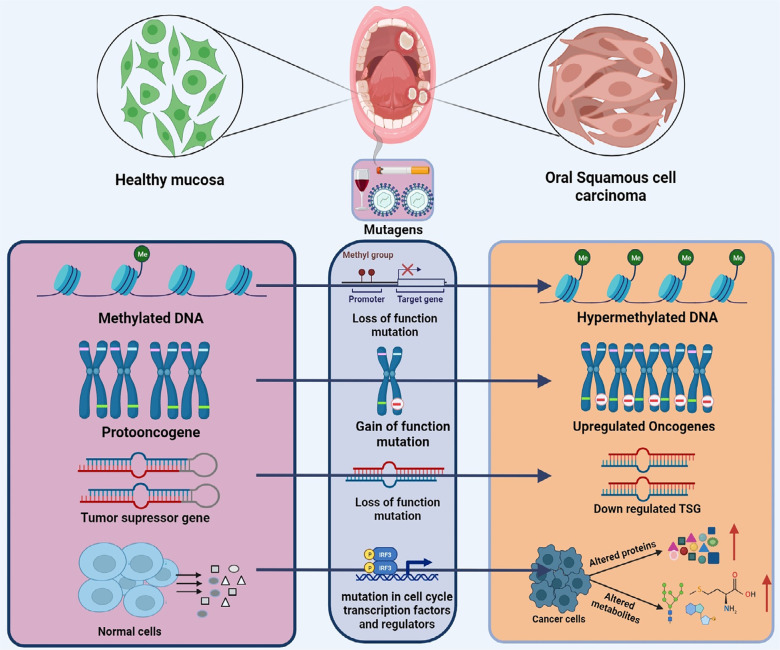
Association of molecular dysbiosis in cancer development-Oral cancer onset stems from various mutations induced by mutagens present in tobacco and alcohol. Numerous molecular dysbiosis, including hypermethylation, gain of function mutation in oncogenes, loss of function mutation in tumor suppressor genes, and several alterations in cell cycle regulators. The cumulative impact of these mutations transforms normal mucosal tissue into oral squamous cell carcinoma.

The development of oral cancer is attributed to numerous mutations, including DNA damage, loss of chromosomal segments, modified methylation, genetic instability, circulating tumor DNA, gene polymorphisms, and epigenetic alterations (Figure 1). DNA can be modified by oxidative stress, hydrolysis, base-pair mismatch, chemicals, ionizing radiation, and ultraviolet (UV) radiation. DNA damage response system disruption may initiate cancer progression. This process involves DNA damage recognition and cell cycle regulation by ATM and ATR enzymes. Impaired ATM/ATR kinase is unable to regulate p53, which inhibits p16 and p52 and regulates the cell cycle, DNA repair, and apoptosis signaling. Mutations in p53, which regulate DNA repair, the cell cycle, and apoptosis, may induce cancer. Early oral cancer is associated with mutant p53 levels. Antibodies against p53 have been detected in the saliva of patients with oral cancer, suggesting that saliva can be a non-invasive method for detecting p53 mutations.^21^

Cancer development is associated with changes in CpG island methylation at gene promoters. Methylation patterns in OSCC and control participants were examined using non-invasive oral rinse and methylation-specific polymerase chain reaction (PCR). OSCC and control participants expressed eight hypermethylated genes differently.^22^ The methylation patterns of salivary MGMT, DCC, CCNA1, TIMP-3, p16, and MINT-31 in healthy individuals and patients with Head and Neck Squamous Cell Carcinoma (HNSCC) were measured using quantitative polymerase chain reaction (Q-PCR). Targeted gene hypermethylation was identified in 54.1% of individuals. Epigenetic changes in oral cancer can be detected by salivary methylation.^23^ Healthy and cancerous cells contain genetic material that differ and can be used as biomarkers. DNA chromosomal abnormalities can cause the spread of cancer. Therefore, examining the abnormalities in ploidy status can aid in the prediction of tumor aggressiveness. Loss of heterozygosity (LOH) occurs when a chromosomal pair loses genetic material and frequently signals early stages of cancer. According to several studies, LOH in tumor suppressor gene regions can cause malignancies.^24^ OSCC often shows recurrent LOH on chromosomes 3, 17, 9, and 13, as shown by multiple studies.^25^

Oral cancer detection is possible using circulating tumor DNA (ctDNA) and gene polymorphisms. Tumor DNA was detected in blood, saliva, and urine samples. Somatic mutations make ctDNAs more selective than cell-free DNA. Both non-metastatic and metastatic tumor cells produce ctDNA and exhibit tumor genetics.^26^ Anticancer treatment steadily reduced ctDNA levels. Low dilution and contamination make salivary ctDNA analyses beneficial. Because saliva touches the tumor, patients with oral cancer have increased levels of ctDNA in their saliva, and previous studies have explored the relationship between salivary TP53 and the dimensions and progression of oral cancer. The prevalence of TP53 mutations in oral rinse is notably higher in cases of oral and oropharyngeal cancers, indicating a clear correlation between saliva and tumor development.^27^ CDKN2A, HRAS, PIK3CA, and TP53 genes were tested in the saliva of patients with HNSCC for somatic mutations using multiplex PCR. A significantly mutated TP53 mutant was also identified.^28^ Salivary DNA from patients with HNSCC and healthy controls were examined for 82 mutations. Of the 11 deregulated genes, PMAIP1 and PTPN1 correlated with the study groups. This finding suggest that the salivary biomarkers examined could be used as prognostic indicators for detecting HNSCC.^29^ Multi-allele gene polymorphisms diversify populations, whereas single-nucleotide polymorphisms (SNPs) control the cell cycle and DNA mismatch repair. Cancer progresses with deregulated SNPs. Researchers have found that oral cancer progression is linked to GSTT1 gene genotype absence.^30^ Yen, et al.^31^ (2008) examined salivary SNPs for the DNA-repairing RAD51 family XRCC gene 14q32.3, which affects cancer signaling. PCR restriction fragment length polymorphisms were used to study XRCC1 (rs1799782), XRCC2 (rs2040639), XRCC3 (rs861539), and XRCC4 (rs2075685) polymorphisms. In XRCC2, only rs2040639 was linked to cancer development. XRCC2 (rs2040639) and XRCC3 (rs861539) presented the most significant differences between cancer and control groups. Various studies have demonstrated that alterations in the DNA present in saliva play a crucial role in the early identification and prognosis of oral cancer due to its direct and close interaction with malignant lesions. Table 1 provides a comprehensive list of the diverse salivary DNA biomarkers associated with mutations, aneuploidy, and LOH.

**Table 1:** Differential expression summary of salivary DNA biomarkers in Oral Cancer

Biomarker	Study population	Saliva partition	Methods	Expression	Sensitivity	Function	References
8-OHdG	90 (30HC, 30 OSCC, 30 OSMF)	Saliva Supernatant	Sandwich ELISA	Upregulated	p<0.0001	Oxidative DNA damage	91
P16INK4A, RASSF1A	83 (40 HC, 43 OSCC)	Salivary rinse	Quantitative methylation specific PCR	Hyper methylation	p<0.001	Tumor suppressor gene	92
TIMP3, PCQAP/MED15	148 (60 HC, 54 OC, 34 OP)	Saliva Supernatant	Quantitative methylation-specific PCR	Hyper methylation	p<0.05 p<0.0001	Tumor suppressor gene	93
Telomerase	300 (100 HC, 100 PML, 100 OSCC)	Salivary rinse	TRAP assay	Upregulated	p<0.001	Reverse Transcriptase enzyme	94
KIF1A, EDNRB	132 (61 HC, 71 HNSCC)	Salivary rinse	Quantitative methylation-specific PCR	Hyper methylation	p<0.0001	Intracellular transport and cell cycle	95
p53 gene codon 63 of exon 4	37 (27 HC, 10 OC)	Saliva Supernatant	DNA sequencing and PCR	Loss of p53 gene codon 63	p<0.05	Tumor suppressor gene	96
DAPK1	174 (31 HC, 143 HNSCC)	Salivary cell pellet	Nested MSP	Hyper methylation	p<0.0001	Cell cycle regulator	97
p16	60 (30 HC, 30 OSMF)	Salivary rinse	RT-QMSP	Hyper methylation	-	Tumor suppressor gene	98
MED15, PCQAP	184 (94 HC, 90 HNSCC)	Saliva Supernatant	Methylation specific PCR	Hyper methylation	p<0.01	Transcription factors	99
Maspin Phospho-Src	38 (19 HC, 19 TC)	Saliva Supernatant	Immunoreactivity assay	Upregulated	p=0.001 p<0.00001	Tumor suppressor, cell-cell adhesion, and proliferation	100
D3S1234, D9S156, and D17S799	50 (10 HC, 40HNSCC)	Saliva Supernatant	Microsatellite analysis	Loss of heterozygosity	p<0.001	Cause Genetic instability	101
MGMT	60 (30 HC, 30 HNSCC)	Saliva Supernatant	Methylation-specific PCR	Hyper methylation	p<0.001	DNA repair pathway	102

HC= Healthy Control; OC= Oral Cancer ;OSCC= Oral Squamous Cell Carcinoma; OSMF= Oral Submucous Fibrosis; OP= Oropharynx; HNSCC= Head and Neck Squamous Cell Carcinoma; PML= Premalignant lesion; 8-OHdG= 8-Hydroxydeoxyguanosine; KIF1A= Kinesin family member 1A; EDNRB= Endothelin receptor type B; TIMP3= Tissue Inhibitor of Metalloproteinases 3; PCQAP = PC2 glutamine/Q-rich-associated protein; PCR= Polymerase Chain Reaction; DAPK1= Death-associated protein kinase 1; OSMF= oral submucous fibrosis; RT-QMSP= Real-Time quantitative methylation-specific PCR; Phospho-Src = Phosphorylated-Src; TC= Tongue Carcinoma; MSP= Methylation specific PCR; Maspin= mammary serine protease inhibitor TRAP= Telomerase Repeated Amplification Protocol; MGMT= methylguanine-DNA-methyltransferase; RAASF1A= Ras association domain-containing protein; MED15= Mediator Complex Subunit 15

The formation of oral cancer involves a wide range of mutations that include DNA damage, changes in chromosomes, patterns of methylation, and variations in genes. Saliva is a valuable diagnostic tool since it can identify genetic abnormalities, such as p53 mutations and LOH, using non-invasive techniques. Moreover, the analysis of DNA in saliva enables the detection of genetic changes and variations linked to the advancement of oral cancer, emphasizing its potential as a diagnostic tool for the early diagnosis and monitoring of the disease.

### Salivary transcriptomics

For decades, RNA was thought to facilitate gene expression by converting DNA sequences into proteins. However, further research has shown that certain RNA molecules, known as non-coding RNA (ncRNA), regulate gene expression by controlling the activation and deactivation of genes. These RNA molecules do not encode proteins and include various types, such as short hairpin RNAs, PIWI-interacting RNAs, short interfering RNAs, small nuclear RNAs, small temporal RNAs, and small nuclear RNAs.^32^ ncRNAs are emerging as key regulators of cancer progression and tumor growth. Their small size and stability in bodily fluids, including blood, urine, and saliva, make them potential candidates for non-invasive diagnostic tests. These tests could revolutionize disease detection, leading to more effective clinical applications and potentially reducing health care costs.

### microRNA in oral cancer

miRNAs are non-coding molecules composed of 19–25 nucleotides, which are generated from pre-miRNAs with the aid of drosha in the nucleus and Dicer in the cytoplasm. These molecules play various roles in cell functions, such as proliferation, division, apoptosis, and immunological responses, as well as in inter-pathogen and host communication. Moreover, miRNA transcripts can act as either tumor suppressors or oncogenes due to their diverse effects. Oral cancer initiation and progression is affected by the abnormal regulation of tumor suppressor and oncogene genes.^33^ It became apparent that circulating miRNAs in saliva were stable, based on their structural assets. MicroRNA (miRNA) analysis was performed on five patients with oral cancer and five study controls using microarray technology to identify differentially expressed miRNAs. The most prominent dysregulated miRNAs were validated using quantitative real-time PCR (qPCR). miR-517b-3p and miR-303b-3p were found to be downregulated in oral cancer, whereas miR-412-3p and miR-512-3p were found to be considerably increased in qPCR analysis. Consequently, a panel of salivary EVs miRNAs can be used as biomarker to identify oral cancer.^34^ The most prevalent salivary miRNAs, miR-146a-5p, miR-205-5p, miR-92a-3p, and miR-124-3p, set oral cancer apart from control participants in a different study with 216 participants, 108 of whom had head and neck squamous cell carcinoma and 108 were healthy controls.^35^

Salivary miRNAs were used to screen for 60 individuals, 30 of whom had OSCC. According to a previous study, patients with oral cancer showed considerable downregulation of let-7c, miR-99a, and miR-100.^36^ Salivary microRNAs (miRNAs) may be promising indicators for oral cancer identification in high-risk populations such as tobacco smokers. Real-time PCR was used to compare salivary miRNA expression between smokeless tobacco users and non-smokers. miR-146a, miR-199a, and miR-155 are considerably upregulated in smokeless tobacco users. These findings indicate that tobacco use may cause epigenetic alterations that lead to oral cancer.^37^ Quantitative real-time polymerase chain reaction, microarray, and next-generation sequencing indicated that salivary miRNA patterns were distinct between patients living with cancer and healthy controls, as listed in Table 2.

**Table 2:** Differential expression of various salivary microRNA biomarkers

Biomarkers	Study population	Saliva partition	Methods	Expression	Function	Sensitivity	References
miR-423-5p	147 (58 HC, 89 OSCC)	Saliva Supernatant (centrifuged at 2600g, 15 minutes)	Microarray and qRT-PCR	Upregulated	Oncogene	p<0.001	103
miRNA-let-7a-5p and miRNA- 3928	230 (80 HC and 150 HNSCC)	Whole saliva	qRT-PCR	Downregulated	Tumor suppressor gene	p<0.0001 p<0.01	104
miR-26a and miR-26b	28 (14 HC, 14 OLP)	Whole saliva	Real-time PCR	Downregulated	Tumor suppressor gene	p<0.001	105
miRNA 31	72 (HC and OPMD)	Saliva Supernatant	qRT-PCR	Upregulated	Oncogene	p=0.01	106
miRNA-124-3p	216 (108 HC, 108 HNSCC)	Whole saliva	miRNA PCR Array and qRT-PCR	Downregulated	Tumor suppressor gene	p=0.002	107
miRNA-191 miRNA-146a	78 (24 HC, 14 OSCC, 14 hOSCC, 13 OSCCr, 13hOSCCr)	Oral brush	Real time PCR	Upregulated	Oncogene	p<0.001	108
miRNA-125a miRNA-200a	60 (15 HC, 15 OSCC, 30 OLP)	Whole saliva	qRT-PCR	Downregulated	Tumor suppressor gene	p<0.0001	109
miRNA-320a	62 (15 HC, 15 OSCC and 32 OLP)	Saliva Supernatant (centrifuged at 3000g, 15 minutes)	RT-qPCR	Downregulated	Tumor suppressor gene	p=0.004	110
miRNA-139-5p	50 (25 HC and 25 TSCC)	Saliva Supernatant (centrifuged at 2600g, 15 minutes)	Microarray and qRT-PCR	Downregulated	Tumor suppressor gene	p=0.006	111
miRNA-21, miRNA-184	100 (20 HC, 40 PMDs, 20 OSCC and 20 RAS)	Saliva Supernatant (centrifuged at 2500g, 10 minutes)	qRT-PCR	Upregulated	Oncogenes	p<0.001	112
miRNA-145	100 (20 HC, 40 PMDs, 20 OSCC and 20 RAS)	Saliva Supernatant (centrifuged at 2500g, 10 minutes)	qRT-PCR	Downregulated	Tumor suppressor gene	p<0.001	112
miRNA-24, miRNA-27b	34 (9 HC, 9 OSCC, 8 OSCC-R and 8 OLP)	Saliva Supernatant (centrifuged at 2600g, 15 minutes)	Nanostring miRNA assay and RT-qPCR	Upregulated	Oncogenes	p<0.05	113
miRNA-136	34 (9 HC, 9 OSCC, 8 OSCC-R, and 8 OLP)	Saliva Supernatant (centrifuged at 2600g, 15 minutes)	Nanostring miRNA assay and RT-qPCR	Downregulated	Tumor suppressor gene	p<0.05	113
miRNA-9	112 (56 HC, 56 HNSCC)	Saliva supernatant	Microarray and qRT-PCR	Upregulated	Oncogenes	p<0.0001	114
miRNA-134	112 (56 HC, 56 HNSCC)	Saliva supernatant	Microarray and qRT-PCR	Downregulated	Tumor suppressor gene	p<0.0001	114
miRNA-148a, miRNA-1250, miRNA-632, miRNA-503, miRNA-323-5p	34 (9 HC, 9 OSCC, 8 OSCC-R and 8 OLP)	Saliva Supernatant (centrifuged at 2600g, 15 minutes)	Nanostring miRNA assay and RT-qPCR	Downregulated	-	p=0.027	113
miRNA-145-5p, miRNA-99b-5p, miRNA-181c, miRNA-197-3p	14 (7 HC and 7 OLP)	Whole saliva	Microarray and RT-qPCR	Downregulated	Tumor suppressor genes	p=0.034 p=0.011 p=0.028 p=0.057	115
miRNA-10b, miRNA-30e	14 (7 HC and 7 OLP)	Whole saliva	Microarray and RT-qPCR	Upregulated	Oncogenes	p=0.008 p=0.089	115

p-value < 0.05 was considered statistically significant.

Salivary miRNAs show potential to act as reliable biomarkers for detecting oral cancer. They can indicate abnormalities in genes that regulate tumor growth and genes that promote the development of cancer. Their unique expression patterns, as evidenced by different molecular approaches, indicate their usefulness in detecting oral cancer, especially in high-risk individuals such as tobacco smokers, emphasizing their involvement in the development of cancer.

### mRNA in oral cancer

Messenger RNAs (mRNAs) are crucial in cells and act as molecular messengers for protein synthesis. They also play a role in ribosome remodeling and utilize approximately 20% of the cellular energy. Several diseases, including cancer, heart disease, and blood disorders can disrupt mRNA translation. Therefore, regulation of mRNA translation is vital for gene expression. The presence of mRNA in body fluids, particularly saliva, which is a non-invasive liquid biopsy, aids in gene expression analysis in both healthy and diseased states.^38^ Salivary mRNA serves as a biomarker for a range of malignancies, including oral, breast, lung, and ovarian cancers. The studies on salivary deregulated mRNA levels in oral cancer are listed in Table 3.

**Table 3:** Expression profile of salivary mRNA in Oral Cancer

Biomarkers	Study population	Saliva partition	Methods	Expression	Sensitivity	Function	References
NAB2, COL3A1	67 (34 HC, 33 OSCC)	Saliva Supernatant	Real-time PCR	Downregulated	p=0.0023	Growth signal response and cell division	116
NUS1, RCN1	51 (10 HC, 41 OSCC)	Whole saliva	qRT-PCR	Upregulated.	p=0.037 p=0.011	Protein modification and Ca binding cell signaling	117
CYP27A1, NPIPB4, MAOB, SIAE	67 (34 HC, 33 OSCC)	Saliva Supernatant	Real time PCR	Downregulated	p=0.0016 p=0.0059 p=0.0009 p=0.0370	Protein modification	116
IL-8	85 (31 HC, 34 OSCC, 20 OLP)	Saliva Supernatant	q-PCR	Upregulated.	p=0.013	Angiogenesis, cell signaling, and replication	118
OAZ 1	125 (25 HC, 25 OSCC, 25 OSCC-R, 25 OLP, 25 I-OLP)	Saliva Supernatant	q-PCR	Upregulated.	p=0.003	Biosynthesis of polyamine	119
SAT	395 (226 HC, 169 OSCC)	Saliva Supernatant	q-PCR	Upregulated.	p<0.002	Transferase enzyme	120
S100P	125 (25 HC, 25 OSCC, 25 OSCC-R, 25 OLP, 25 I-OLP)	Saliva Supernatant	q-PCR	Upregulated.	p=0.003	Calcium-binding protein	121
DUSP1	125 (25 HC, 25 OSCC, 25 OSCC-R, 25 OLP, 25 I-OLP)	Saliva Supernatant	q-PCR	Upregulated.	p<0.001	Cell signaling and protein modification	121
IL1B H3F3A	64 (32 HC, 32 OSCC)	Saliva Supernatant	Microarray and qPCR	Upregulated.	p<0.001	Inflammation, cell proliferation, cell signaling, and H3F3A in the DNA binding process	122
MAP2K3, B2M	64 (32 HC, 32 OSCC)	Saliva Supernatant	Microarray and qPCR	Upregulated.	p<0.001	Protein modification and antiapoptotic	122
Endothelin-1	16 (HC, OSCC)	Saliva Supernatant	RT-PCR	Upregulated.	p<0.001	Vasoactive peptide	123

p-value < 0.05 was considered statistically significant.

HC= Healthy Control; OSCC= oral squamous cell carcinoma; qRT-PCR= quantitative real time polymerase chain reaction; OSCC-R= OSCC recurrence; OLP= oral leukoplakia; NAB2= NGFI-A binding protein 2; NPIPB4= nuclear pore complex interacting protein family, member B4; COL3A1= collagen, type III, alpha 1; MAOB = monoamine oxidase B; qPCR= Quantitative real-time PCR; MAP2K3= Mitogen-activated protein kinase 3; B2M=beta2microglobulin; IL1B= Interleukin 1 beta; H3F3A= H3 histone, family 3A; SIAE= sialic acid acetyltransferase; IL-8= Interleukin 8; S100P= S100 calcium binding protein P; DUSP1= Dual specificity phosphatase 1; OAZ 1= Ornithine decarboxylase antizyme 1; NUS1= nuclear undecaprenyl pyrophosphate synthase 1; RCN1= reticulocalbin 1; SAT= Spermidine/spermine N1-acetyltransferase.

### Salivary proteomics

Analysis of the salivary proteomes of individuals with oral cancer is expected to be a promising method for identifying new biomarkers for the disease because oral cancer cells are deeply involved in the salivary environment. Proteins play a crucial role in the survival, development, and division of cells in various biological processes. The disruption of proteins can impair the fundamental functioning of cells, leading to various forms of cancer. The identification of changes in protein expression allows for early cancer detection and prediction.^39^ Numerous studies have investigated protein levels in the saliva of patients with oral cancer.^40^Table 4 shows data on the notable alterations in crucial salivary proteins in oral cancer.

**Table 4:** List of oral cancer protein biomarkers and their expression levels

Biomarker	Study population	Saliva partition	Method	Expression	Sensitivity	Reference
LDH	100 (HC, OLP, OLRs, and OSCC)	Saliva Supernatant (centrifuged at 2600g, 15 minutes)	Spectrophotometry	Upregulated	p<0.02	81
IL-8 IL-1β LGALS3BP	117 (HC, OSCC, PMOD, and under treatment)	Saliva Supernatant	ELISA	Upregulated	p<0.0006 p<0.0001	124
IL-6	44 (HC and OC)	Saliva Supernatant	Immuno-fluorescence	Upregulated	p<0.05	125
MMP-1	1160 (HC, OPMD-I, OPMD-II, and OSCC)	Saliva Supernatant	ELISA	Upregulated	p<0.001	126
MMP-9	88 (HC, OSCC and OPMD)	Saliva Supernatant	ELISA	Upregulated	p<0.001	127
IL-17A IL-17F TNF-α	71 (HC, Differentiated site and grade tumor)	Saliva Supernatant	ELISA	Upregulated	p<0.001 p<0.01 p<0.01	128
CYFRA21-1	35 (OPMD, OSMF and OSCC)	Saliva Supernatant	ELISA	Upregulated	p=0.01	129
TNF- α IL-6	60 (HC, OBFOL)	Saliva Supernatant	ELISA	Upregulated	p=0.039 p<0.0001	130
L-Fucose	85 (HC, OPMD and OC)	Saliva Supernatant	Spectrophotometry	Upregulated	p<0.001	131
MUC1	30 (HC, oral premalignant and OSCC)	Saliva Supernatant	ELISA	Upregulated	p<0.001	132
Hsp27	45 (HC and OLP)	Saliva Supernatant	ELISA	Upregulated	p<0.001	133
						
Hsp90 L-Fucose	90 (HC, PMOD, and OC)	Saliva Supernatant	ELISA	Upregulated	p<0.001	134
Sialic acid (FSA and PBSA)	96 (HC, tobacco chewers and OSCC)	Saliva Supernatant	Spectrophotometry	Upregulated	p<0.001	135

p-value < 0.05 was considered statistically significant.

LDH= Lactate Dehydrogenase; IL= Interleukin; LGALS3BP = galectin-binding protein; MMP= Matrix metallopeptidase; TNF= Tumor Necrosis Factor; CYFRA= Cytokeratin fragment; MUC= Mucin; Hsp= Heat shock protein; FSA= Free sialic acid; PBSA= Protein-bound sialic acid; HC= Healthy Control; OC= Oral Cancer; OLP= Oral Leukoplakia; OLRs= Oral lichen planus; OSCC= Oral Squamous Cell Carcinoma; PMOD=potentially malignant oral disorders; OPMD= Oculopharyngeal muscular dystrophy; OBFOL= oral benign fibro-osseous tumors; IL= Interleukin; MMP= Matrix metalloproteinase; PBSA=Protein-bound sialic acid; FSA= Free sialic acid.

Tetranectin, a molecule that binds to plasminogen and is implicated in tumor metastasis, was found to show notably reduced levels in a study of 15 participants, including healthy individuals and those with primary and advanced OSCC. This reduction suggests tumor metastasis or advanced tumors.^41^ Salivary albumin protein, an ultrafiltrate of serum albumin, is increased in various diseases, and researchers have shown that patients with OSCC have higher salivary albumin levels than healthy individuals.^42^ Survivin, an inhibitor of cell death, is a prospective biomarker for various cancers including OSCC. Salivary survivin levels were assessed in patients with cancer and healthy individuals; those with OSCC had considerably higher levels, suggesting that salivary survivin may be used as a diagnostic biomarker.^43^ Salivary proteases play a crucial role in the development of oral cancer. Feng et al. found differences in protease levels between healthy individuals and those with OSCC, identifying several upregulated proteases, including MMP 1–3, 10, and 12, ADAMST13, ADAM9, cathepsin V, Kallikrein5, and Kallikrein7.^44^

Amylase in saliva breaks down starch secreted by the parotid glands. Salivary amylase levels vary among healthy individuals, patients with oral cancer, and those undergoing treatment, with lower levels occurring during cancer therapy.^45^ According to a previous study, the OSCC group had lower salivary amylase levels than the healthy control group (p=0.12). Awasthi et al. evaluated CYFRA21-1, CA19-9, lactate dehydrogenase (LDH), total protein, and amylase levels in 64 healthy, premalignant, and patients with OSCC. The levels of all biomarkers, except amylase, increased considerably.^46^ In conclusion, OSCC and premalignant lesions mostly showed low amylase levels. Conversely, the salivary amylase concentration was considerably lower (p<0.001) in healthy individuals than in patients with oral cancer and those undergoing treatment. Amylase levels decreased until week 3, showing increases from weeks 3 to 6.^47^

Cytokines are proteins that regulate the immune response, cell growth, and blood vessel formation. They can be pro-inflammatory, including IL-1β, IL-6, and TNF-α, or anti-inflammatory, including IL-1, IL-4, and IL-10. Salivary cytokine levels increase in patients with oral cancer, affecting the oral lesions.^18,48^ A study of 90 patients found higher salivary TNF-α levels in oral leukoplakia and OSCC groups than in healthy controls, suggesting that TNF-α is a biomarker for oral dysplasia.^49^ Another study compared IL-8 levels between healthy controls and OSCC patients. Patients with OSCC show higher IL-8 levels, indicating their potential as biomarkers of OSCC.^50^ Apart from these altered salivary biomarkers in oral cancer, other biomarkers, such as cyclin D1, Ki 67, defensin-1, profiling-1, catalase, annexin-1, calcyclin, and SCCA-2 have also been reported in oral cancer.^51^

Therefore, the examination of salivary proteomes shows potential for identifying new biomarkers for oral cancer, given the complex interaction between oral cancer cells and the salivary environment. Patients living with oral cancer have shown significant changes in important proteins, including tetranectin, albumin, survivin, proteases, and amylase, suggesting that these proteins could be useful for diagnosing the disease. Moreover, alterations in the levels of cytokines in saliva, including TNF-α and IL-8, emphasize the importance of salivary biomarkers in the identification and prediction of oral cancer.

### Salivary metagenomics

Metagenomics studies the genomes of multiple microorganisms in complex communities. The oral microbiome comprises various bacteria that form symbiotic relationships in the mouth. It is the second largest component of the gut microbiota, with over 700 bacterial species belonging to 12 phyla and 185 genera, which vary among individuals based on lifestyle and physiological conditions. The 12 phyla were designated as *Firmicutes*, *Proteobacteria*, *Fusobacteria*, *Chlamydiae*, *Bacteroidetes*, *Actinobacteria*, *Spirochaetes*, *SR1*, *Chloroflexi*, *Synergistetes*, *Gracilibacteria*, *and Saccharibacteria*. These microbial communities belong to 70% culturable and 30% non-culturable species.^52^ In addition to the core microbial community, the oral microbial community also exhibited a spatially and temporally differential pattern. The altered dynamics are influenced by factors such as the disease progression of oral cancer, genetic mutations, and changes in pH levels, smoking, alcohol consumption, pathogen infection, and tooth decay. The examination of altered oral microbiota reflects disease progression in the oral cavity.^53^

Recent technologies have been developed to study the oral microbiota, including culture and microscopy, DNA microarray, PCR, 16S rRNA, and next-generation sequencing. A correlation was found between oral cancer progression and five microbial genera, namely *Enterococcus*, *Peptostreptococcus*, *Bacillus*, *Slackia*, and *Parvimonas*, suggesting their potential as predictive and diagnostic indicators for OSCC detection.^54^ Significant variations in *Prevotella melaninogenica*, *Streptococcus mitis*, and *Capnocytophaga gingivalis* were identified in another study that compared the oral microbiota of patients with OSCC to that of healthy controls.^55^ Microbial diversity in the oral cancer, oral leukoplakia, and healthy groups were analyzed using Illumina sequencing. Significant differences were found between the healthy and oral cancer groups but not between the oral lichen planus (OLP) and oral cancer groups. The most differential pattern between the oral cancer and the control group included *Prevotella*, *Streptococcus*, and *Salmonella. Fusobacterium nucleatum*. and *Porphyromonas gingivalis* has been suggested to play a role in releasing inflammatory cytokines, promoting cell proliferation and invasion in oral cancer, thus it is thought to be involved in cancer metastasis.^56^ *P. gingivalis* contributes to TNF-α and metalloproteinase production and inhibits p53, whereas *F. nucleatum* secretes lipopolysaccharides and cytokines that are linked to cancer development.^57^

Yang, et al.58 (2018) found increased Fusobacterium and decreased *Streptococcus*, *Actinomyces*, *Porphyromonas*, and *Haemophilus* in saliva as cancer progressed from stages 1 to 4. Several other studies have shown that *Firmicutes* (*Streptococcus*) and *Actinobacteria* (*Rothia*) are significantly decreased in patients with cancer compared to those in the non-cancerous group.^59^ *Firmicutes*, specifically *Streptococcus*, contributes to DNA damage via acetaldehyde production, leading to lipid peroxidation and reduced *Lactobacillu*s spp, creating a tumor-friendly environment. *Actinobacteria* play a role in periodontal diseases, plaque formation, and caries. Alcohol and tobacco use affect the oral microbial diversity. Smokers and alcoholics were compared to the oral microbiome of nonsmokers. *Capnocytophaga* and *Prevotella* were upregulated, whereas *Staphylococcus*, *Peptostreptococcus*, and *Granulicatella* were downregulated in smokers compared to those in the control group.^60^

Metagenomic studies show the oral microbiome’s complex composition and dynamics, which may affect oral cancer detection and progression. Emerging technologies can identify oral cancer-associated microbial genera, demonstrating their predictive and diagnostic potential. Microbial variety underscores their role in inflammation and cancer metastasis, emphasizing the oral microbiota’s role in mouth cancer formation.

### Salivary metabolomics

Metabolomics, an advanced omics technique, is associated with abnormal gene expression, drug revelation, and exposure to carcinogens. Different metabolite patterns have been examined using modern technology to detect oral, periodontal, pancreatic, and breast cancers.^61^ These have included metabolomic analyses of human body fluids, cells, and tissues for metabolic intermediates. Modern metabolomics can quantify disease progression biomarkers using several sophisticated technologies including time-of-flight mass spectrometry (TOF-MS), capillary electrophoresis, and Nuclear Magnetic Resonance (NMR).^38^ In addition to its core function, saliva contains metabolites that can detect oral cancer. Wei, et al.^62^ (2011) compared salivary metabolites from OSCC, OLK, and controls using ultra-performance liquid chromatography and TOF-MS. Compared to the OLK and control groups, the OSCC group had significantly higher salivary levels of n-eicosanoic acid and lactic acid, and lower salivary levels of GABA, phenylalanine, and valine. Enhanced glycolysis and anaerobic respiration produce lactic acid, which is a cancer marker. Increased energy utilization causes cancer cells to undergo anaerobic glycolysis, creating hypoxia. Valine provides energy, whereas phenylalanine signals and generates proteins. Lohavanichbutr, et al.^63^ (2018) found that four salivary metabolites, namely proline, glycine, citrulline, and ornithine, were significantly altered in the healthy and OSCC groups.

Sugimoto, et al.^64^ (2010) performed capillary electrophoresis-mass spectrometry to compare the levels of OSCC and control salivary metabolites. Study groups discovered 28 metabolites. Phyroline, hydroxycarboxylic acid, choline, tryptophan, threonine, carnitine alpha-aminobutyric acid, and phenylalanine showed significant p-values. Combined biomarkers can differentiate between oral cancer and healthy individuals. Another study examined the salivary metabolites of patients with OSCC and healthy controls, namely L-carnitine, betaine, pipecolinic acid, and choline, using ultraperformance LC-MS. Patients with OSCC showed highly elevated betaine, pipecolinic acid, and choline levels, while under expressing L-carnitine (96.7% specificity, 100% sensitivity).^65^ Another study used hydrophilic interaction chromatography and performed metabolomic analysis of OSCC to detect salivary metabolites, including propionylcholine, S-carboxymethyl-L-cysteine, phytosphingosine, sphinganine, and N-acetyl-L-phenylalanine.^66^ Ohshima et al. compared salivary metabolites in OSCC and control groups and found 25 differentially expressed metabolites including hydroxyphenyl acetic acid, choline, and 2-hydroxy-4-methylvaleric acid (p<0.001).^67^ In tumor cells, choline is metabolized to phosphocholine, followed by its oxidation to betaine.

Sridharan, et al.^68^ (2019) used LC-MS and mass Hunter profiles to examine salivary metabolites in OSCC, OLP, and healthy controls. The results demonstrated significant (p<0.05) upregulation of inositol 1,3,4-triphosphate, d-glycerate-2-phosphate, 1-methylhistidine, 2-oxoarginine, 4-nitroquinoline-1-oxide, pseudouridine, sphinganine-1-phosphate, and norcocaine nitroxide, as well as downregulation of ubiquinone, estradiol valerate, neuraminic acid, and l-homocysteine acid metabolites. Metabolomic studies have shown that various salivary metabolites exhibit variable expressions. These metabolites may serve as early markers for diagnosing oral cancer either independently or in combination with other metabolites.

### Salivary exosomes

Exosomes are nanoparticles with double-layered membranes that originate from the endosomal pathways and bodily fluids. Salivary exosomes contain molecular cargo, support physiological functions, and serve as biomarkers of physiological changes.^69^ Salivary exosomal materials containing tumor-derived nucleic acids, proteins, and metabolites are stable and suitable for investigating oral cancer. Exosomal content controls immunomodulation and tumor enhancement and indicates microenvironmental fluctuations.^70^ Studies have shown that salivary exosomes in patients with oral cancer are larger, more numerous, and more diverse.^71^ In addition, Fourier-transform infrared spectroscopy indicated that salivary exosomes and their contents are protected from cellular nucleases.^72^ MicroRNAs (miRNAs), proteins, and DNA in salivary exosomes are linked to cancer progression. Salivary exosomal miRNAs are non-coding RNAs that function as tumor suppressors and oncogenes under normal physiological conditions. miR-412-3p, miR-512-3p,^73^ miR-1246, miR-342-3p, and miR-24-3p^74^ showed differential patterns and could be used as diagnostic biomarkers. Exosomal miRNAs promote cancer cell migration via the MAPK cell signaling system, upregulate oncogenic genes, and downregulate tumor suppressor genes.

Salivary exosomal proteins demonstrated that patients with oral cancer and healthy individuals have varied expression of MUC5B, HPA, LGALS3BP, A2M, IGHA1, GAPDH, and PKM1/M2. This robust proteomic analysis can distinguish OSCC from the controls with 90% accuracy.^75^ Oral cancer exosomes have higher levels of CD63, a 25kDa salivary protein that regulates cell invasion and migration, whereas in an exosomal salivary protein study, oral cancer and healthy groups exhibited elevated CD63, as well as downregulated CD9 and CD81.^76^ As described by the biological role of exosomes in oral cancer progression, the salivary exosomal content can be used as a prognostic and diagnostic biomarker. These nanoparticles are incredibly informative as they are safely packed and delivered to the extracellular environment and show advantages over salivary complexity. Therefore, salivary exosomes have emerged as new tools for the diagnosis of oral cancer.

### Common biomarkers from invasive and non-invasive diagnostic tools

The human body consists of several tissues and fluids, including saliva, blood, cerebrospinal fluid (CSF), tears, perspiration, and urine. They show distinct purposes and consist of proteins, electrolytes, hormones, and metabolites that are crucial for health. Blood, saliva, and urine are important for the diagnosis of diseases, and the blood is particularly useful for detecting changes in the body. Tissue biopsy, blood biopsy, exfoliated cell cytology, PET, and CT-SCAN are the key cancer diagnostic methods; however, they have limitations in late-stage diagnosis with painful procedures. Saliva is a non-invasive tool and holds potential to be an alternative to invasive procedures. This fluid is widely available, simply collectible, and storable, especially beneficial in oral cancer, as it is in direct contact with the lesion.^77^ Several earlier studies have shown that saliva carries the filtrate of plasma and serum and alters cell-free and cell-based tumor cells undergoing necrosis and apoptosis. Hence, saliva can rise above the invasive methods of diagnosis. Several studies have compared and found various significantly altered common biomarkers in the same study cohort using non-invasive and invasive methods of oral cancer diagnosis.

Dadhich, et al.^78^ (2014) discovered that individuals with oral cancer and premalignant lesions had significantly upregulated (p<0.0005) sialic acid in their serum and saliva samples as compared to healthy controls. The glycoprotein sialic acid can easily be detected in saliva and has been associated with carcinogenesis. Sartini, et al.^79^ (2012) reported that patients with OSCC had considerably higher nicotinamide N-methyltransferase levels (p<0.0001) in both saliva and tissue than the healthy group. The study concluded that selected enzymes could be used as early biomarkers for OSCC using a non-invasive sample type. In another study, Bhat, et al.^80^ (2017) found a significant (p<0.001) decrease in the antioxidant ascorbic acid in the saliva and serum samples of healthy individuals, those with potentially malignant conditions, and patients living with oral cancer. Salivary antioxidant levels can reliably and non-invasively detect OSCC changes reliably and non-invasively.

Gholizadeh, et al.^81^ (2020) explored the patterns of salivary and plasma lactate dehydrogenase (LDH) levels in healthy controls, OSCC, oral lichen planus, and oral lichenoid eruption. LDH concentrations were considerably higher in patients with OSCC, which is crucial for the maintenance of a healthy oral mucosa. Thus, salivary LDH levels indicate mucosal epithelial damage and metastasis. Dineshkumar, et al.^82^ (2016) observed that the malignant and OSCC groups had higher saliva, serum IL-6, and pro- and anti-inflammatory cytokine levels than the groups composed of healthy patients and those with premalignant lesions. The identified biomarkers did not differ significantly across non-invasive and invasive sample types. Additional research has compared salivary and serum levels of IL-10, VEGF, TNF alpha, and TNF-β cytokines in healthy individuals. In the OSCC group, saliva and serum showed significantly higher levels of all the potential biomarkers. Thus, salivary cytokines may serve as prognostic biomarkers for oral cancer detection and prognosis biomarkers.^83^ Other cytokines found in the saliva and serum include IL-8, IL-1β, and eotaxin.^84^ Saliva and serum tetranectin levels were investigated in healthy controls and patients with OSCC at early and late metastatic stages. Saliva and serum tetranectin levels were considerably lower (p=0.007) in patients with OSCC and subsequent metastases. Matrix metalloproteinases (MMPs) are enzymes responsible for extracellular matrix degradation. MMP levels have been studied in the saliva and serum of OSCC and healthy groups, and they were found to be significantly upregulated in both saliva and serum samples of the OSCC group.^85^

**Figure 2:**
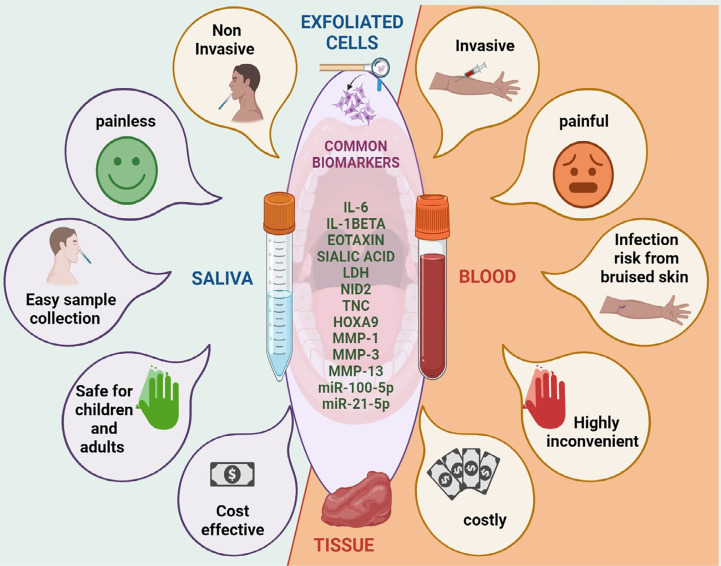
Comparison of non-invasive and invasive biomarkers-Comparison of non-invasive and invasive biomarkers: The utilization of non-invasive approaches such as saliva and oral exfoliated cells conquer the drawbacks associated with invasive methods such as tissue biopsy and fine-needle aspiration cytology (FNAC). The painless, convenient, and safe nature of non-invasive sample collection methods provides an advantage over the painful, inconvenient, and risky aspects associated with invasive methods.

Saliva miRNA profiling is a promising non-invasive oral cancer diagnosis approach. Cao, et al.^86^ (2018) conducted methylation-specific PCR to compare miRNA promoter methylation in patients with HNSCC and controls. They identified seven methylated genomic loci encoding miRNAs (mgmiRNA) that were altered in saliva and tissues, including mgmiR9-1, mgmiR124-3, mgmiR124-2, mgmiR124-1, mgmiR137, mgmiR129-2, and mgmiR148a. According to this study, a panel of salivary mgmiRNAs may help diagnose HNSCC.^86^ Systematic studies have evaluated and compared diverse oral cancer biomarkers using invasive and non-invasive sample collection since they carry common biomarkers (Figure 2).

Overall, the various tissues and fluids found in the human body provide essential information about health and disease. The diagnostic efficacy of saliva can be considered remarkable within the realm of non-invasive diagnostic procedures. Saliva shows promise as a non-invasive diagnostic tool for detecting oral cancer. Saliva analysis demonstrates shared biomarkers with invasive techniques, including sialic acid, LDH, cytokines, and miRNAs, highlighting its diagnostic effectiveness and promise as a substitute for invasive treatments.

### Validation and Demerits to be taken care of for saliva use

Saliva is a valuable medium for investigating functional indicators associated with disease development because of its non-invasive nature, accessible sample collection, processing, and portability. However, validating small amounts of molecular biomarkers in saliva requires more sophisticated technologies than in other bodily fluids. Various methods have been established for this purpose, but saliva shows limitations such as potential cross-contamination and variability in composition. This section describes the validation and drawbacks of using saliva as the diagnostic medium.

### Salivary validation points to be concerned

For saliva sample collection and processing to yield accurate and reliable results, the following guidelines should be followed:

- To prevent contamination, patients should refrain from eating or drinking for at least two hours before to sample collection.
- Sample collection should be taken from 8 a.m. to 10 a.m. to maintain the circadian rhythm, which can affect biomarker levels.
- Saliva samples should avoid mucous contamination, which can affect the analysis.
- Saliva samples should be processed and stored as soon as possible to avoid biomarker degradation.
- When processing saliva samples, they should be stored at room temperature for immediate processing, 4°C for processing samples within 3–4 h, and −80°C for processing samples after several days to a month.
- The number of freeze-thaw cycles should be reduced in the sample because they can cause deterioration of organic contents.
- The sample should not be exposed to air, aiming to prevent RNA degradation caused by RNAse activity.
- Validation of salivary biomarkers using advanced technology should employ mass spectrometry, polyacrylamide gel electrophoresis, and matrix-assisted laser desorption ionization-time of flight (MALDI-TOF) for conventional protein analysis, as well as advanced polymerase chain reaction and microarray for DNA and RNA elucidation. Gas chromatography-mass spectrometry (GC-MS) and microbiomes should be analyzed with next-generation sequencing.^87^

### Demerits of Saliva

- Salaried biomarkers are less concentrated than those in other biofluids, making detection difficult, even with advanced technologies.
- Accurate saliva collection requires sensitive devices.
- The process of biomolecule transfer between blood and saliva is poorly understood, complicating salivary biomarker studies.^3^

## Conclusion

Oral cancer includes cancers that affect the oral cavity, with OSCC being the most common subtype. OSCC is an epithelial neoplasia with high morbidity and mortality rates and low five-year survival rate. Several factors, including late-stage detection and lack of adequate knowledge regarding early alterations, are responsible for most of the health burden associated with oral cancer. These factors, in turn, lead to the progression of oral cancer. Alterations in genetic and epigenetic material are among the early processes that can contribute to the establishment of metastatic cancer, which is both complicated and subtle. These changes are caused by several factors, including mutations, radiation, dental caries, alcohol consumption, and tobacco consumption.^88^ The incidence of oral cancer is ten times more prevalent among individuals who smoke tobacco products. According to the findings of several scientific studies, tobacco consumption is directly linked to the development of mouth cancer. This is due to the fact that tobacco contains 60 known carcinogenic compounds, which contribute to the development of oral cancer. Screening for altered biomarkers is essential for detecting oral cancer. The current diagnostic methods for oral cancer, including tissue biopsy and fine needle aspiration cytology, show limitations such as invasiveness, cost, and the need for trained personnel. Saliva has been used for the screening and diagnosis of various diseases, including drug abuse, viral infections, autoimmune disorders, oral cancer, and periodontal diseases. The use of saliva, which is non-invasive, holds potential to serve as an alternative to invasive treatments. This body fluid is easily collected, accessible, and stored, in addition to being in immediate contact with oral cancer lesions. Saliva shows a molecular profile that can be used to detect and screen for cancer-associated mutations, as it contains all biomarkers. Thus, it allows the reading of diagnostic areas, including genomics, transcriptomics, proteomics, metabolomics, and metagenomics. The early diagnosis approach assists in treating cancer effectively and on time, helping combat the critical features of cancer. Screening tobacco consumers for early biomarkers might play a significant role. The application of salivaomics, which offers a holistic approach, enables the use of salivary biomarkers in the diagnosis and treatment of oral cancer and other disorders. Non-invasive saliva-based biosensors offer a promising diagnostic approach. These biosensors demonstrate high sensitivity, affordability, and painless detection, making them advantageous for early oral cancer detection. Studies have used various biomarkers and materials, such as salivary CYFRA-21-1 and L-cysteine capped lanthanum hydroxide nanostructures, for biosensor development, with promising results.^89,90^ Additionally, saliva-based biosensors show potential for detecting other health conditions, including kidney disorders and periodontal health issues, further highlighting their versatility and potential impact in clinical practice.

There are few limitations associated with saliva, such as the possibility of cross-contamination and fluctuations in composition; nonetheless, the benefits of saliva as a non-invasive diagnostic source for oral cancer outweigh these drawbacks. More research needs to be conducted using non-invasive methods for oral cancer detection at an early stage. In this context, saliva may represent a key diagnostic medium and our knowledge on it needs to be expanded. Further research is required to establish protocols for the diagnosis and treatment of oral cancer, including metastatic progression, to ensure its success. This will pave the way for the development of saliva-based biosensors, which will enhance the early identification of oral cancer in vulnerable populations, such as tobacco smokers.
